# Modification of Polymer Based Dentures on Biological Properties: Current Update, Status, and Findings

**DOI:** 10.3390/ijms231810426

**Published:** 2022-09-09

**Authors:** Durratul Aqwa Mohd Farid, Nur A’fifah Husna Zahari, Zulfahmi Said, Mohd Ifwat Mohd Ghazali, Lee Hao-Ern, Syazwani Mohamad Zol, Sami Aldhuwayhi, Muhammad Syafiq Alauddin

**Affiliations:** 1 Faculty of Dentistry, Universiti Sains Islam Malaysia, Kuala Lumpur 55100, Malaysia; 2 Department of Basic Sciences and Oral Biology, Faculty of Dentistry, Universiti Sains Islam Malaysia, Kuala Lumpur 55100, Malaysia; 3 Smart Manufacturing and Advanced Renewable Technology Research Group, Faculty Science and Technology, Universiti Sains Islam Malaysia, Nilai 71800, Malaysia; 4 Department of Prosthodontics, College of Dentistry, Majmaah University, Al-Majmaah 11952, Saudi Arabia; 5 Department of Conservative Dentistry and Prosthodontics, Faculty of Dentistry, Universiti Sains Islam Malaysia, Kuala Lumpur 55100, Malaysia

**Keywords:** polymers, PMMA, denture, denture base, prosthodontics

## Abstract

Polymers remain an integral part of denture fabrication materials, specifically polymethylmetacrylate (PMMA). PMMA has been extensively used, particularly in construction as a denture base material. Nonetheless, various challenges, including microbial threats in the form of candidiasis occurrence, still remain a biological challenge to denture wearers. The present article comprehensively reviews the biomodifications introduced to denture components, in particular denture base material, to improve the overall biological properties, together with physical, mechanical, structural integrity, and optical properties. In addition, fundamental information specifically to PMMA as a conventional denture base material and the causative aetiological microbial agents for biological threat to dentures are explored.

## 1. Introduction

Dentures were developed thousands of years ago, starting in 3000 BC. They were developed in Egypt, known as the earliest country to develop medical and prosthetic devices. Research has found that the first dental prosthesis was registered in Egypt in 2500 BC [1]. The evolution of complete denture bases was found in the 16th century by the construction of a complete set of upper and lower dentures in Switzerland that was made from human bone, which are made from the curve-molded bone layouts, coarsely cut from a bull’s femur and intertwined at their furthest back points to frame a pivot. In the 18th century, dentures made from metals and other natural products were introduced, followed by the construction of porcelain in the late 18th century [2].

Polymer, particularly polymethyl methacrylate (PMMA) has been extensively used as a denture base material mainly due to its cost-effectiveness, simple fabrication and manipulation, easier maintenance and repair process, low density, satisfactory physical and mechanical properties, and optical properties almost similar to natural human gum. Initially, Rohm and Hass established the use of PMMA in sheet design, whereas Nemours pioneered the use of PMMA in powder structure. Dr. Walter Wright then utilized PMMA as a denture base material, which was employed as a prosthesis component for 10 years [3].

Nonetheless, various intraoral biological threats may occur to denture wearers. Denture stomatitis (DS), also known as chronic erythematous candidiasis, is an infection of oral mucosa that occurs conventionally beneath the dental prosthesis and most commonly in the palatal area [4]. The clinical manifestations of denture stomatitis can be seen as the appearance of mucosal inflammation, erythema and hyperplasia, a burning sensation of the affected site and discomfort and a change in taste perception.

The denture stomatitis is mainly caused by microbes and fungus on the denture. A prolonged period of denture wearing could promote the flourishing of the microbes and fungus. Ergo, it is essential that the dentures carry antibacterial properties to inhibit the growth of harmful microbes. Although PMMA has superior mechanical properties, its biological properties can still be improved. One of the ways that researchers have explored enhancing this material is through modifying its resin matrix by incorporating nanoparticles and micro filler into the material [5].

There was limited literature that highlighted the modifications introduced to reduce an array of issues to the denture base that might have a negative impact to denture wearers. This study aims to summarize recent modifications on conventional and digital dentures to improve the bioactivity and physical and mechanical response on denture base area intraorally.

## 2. Methodology

A literature search was performed on electronic databases dated up to 28 February 2022, including PubMed/Medline, OVID, and Web of Science, using the following keywords: ‘biology denture’, ‘bioactivity denture’, ‘modification denture’, ‘digital denture biology’, among others. Documents published in English were selected, and the articles were further screened to identify their relevance to this review. Articles included were published approximately 5 years ago until the recent year (2017 onwards until 2022). The articles selected were primarily in vitro studies and/or analytical experimental research papers. The published selected articles must consist of a primary result, with biological properties outcome. Articles including case reports or maxillofacial prostheses, such as obturators, are not included. This narrative review briefly discusses the materials used to modify denture parts, particularly the denture base. The article also navigates on methodology for blending or synthesizing the material if any of those methods are described in the articles selected. Further recommendation from each paper was also briefly acknowledged as part of possible future advancement in the field.

Generally, the studies selected were considered an integral part of the body of the discussion due to the cohesiveness and consistency of reporting on biological activities and the vast majority also reported on other aspects, such as physical and mechanical investigation. The studies showed a potentially exciting additional benefit of PMMA in terms of biological, mechanical, and structural integrity as well as strength and durability, upon modification with other organic or inorganic substrates. These findings will hopefully further consolidate and improve the versatility of PMMA in dentistry, particularly as prosthetic materials [6]. In future works, comparison with other available polymer commonly used in dentistry are desirable. It might also act as a platform for more investigations on hybrid or newer materials.

## 3. Polymethyl Methacrylate (PMMA)

**Figure 1:**
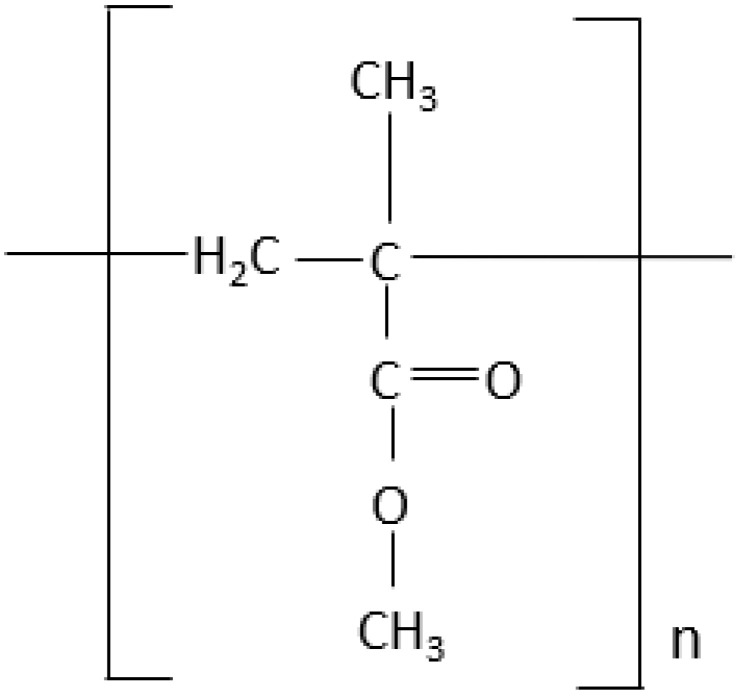
The structure of PMMA.

**Figure 2:**
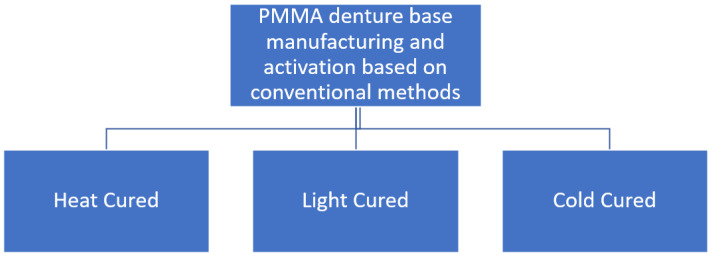
Denture base fabrication conventionally based on PMMA material.

PMMA is essentially an essence derived from an ester of methacrylic compound (CH_2_=C[CH_3_]CO_2_H), as in Figure 1, and it is a synthetic resin polymer produced by polymerization of methyl methacrylate (C_5_O_2_H_8_) to polymethylmethacrylate (C_5_O_2_H_8_)n. Polymerization is conventionally under the influence of an initiator and activated by free radicals, chemically or under other potential sources, such as light and heat. The denture base PMMA material fabrication and activation can be divided into other groups, as shown in Figure 2.

**Figure 3:**
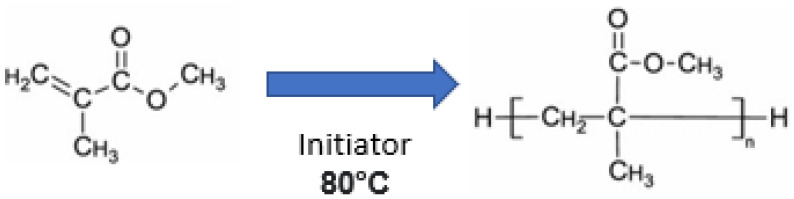
Chemical reaction of heat curing process [7].

The denture base polymers can be classified based on the polymerization activators, such as heat cure, light cure, or cold cure, which can differ in the type of polymerization activation and compositions. In the heat-cured type of PMMA, the polymerization reaction begins when the powder containing PMMA is mixed together with benzoyl peroxide initiator, a plasticizer (dibutyl phthalate) and liquid composed of methyl methacrylate (MMA) monomer, and ethylene glycol dimethacrylate that act as a cross-linking agent with hydroquinone as an inhibitor. Theoretically, the polymerization heat curing process is activated by heat as shown in Figure 3. The chemical reaction in this polymerization process of acrylic resin is when the process conversion of the monomer (MMA) to the polymer (PMMA) become a mixture of the two is subjected to heat by water bath to activate the initiator to dissociate into carbon dioxide (CO_2_) and generate free radicals when heated.

Meanwhile, for the light curing process, these components included a photo initiator system, an acrylic copolymer and microfine silica as fillers and a urethane dimethacrylate matrix for the light curing process. The photo initiator is also activated by the same visible light source that was used for cold curing light as activated composite. S unique high energy light source was employed to provide soft sheets that had already been combined for final polymerization. As a result, individuals who are allergic to methyl methacrylate monomer can use the light activated denture base materials as an alternative.

**Figure 4:**
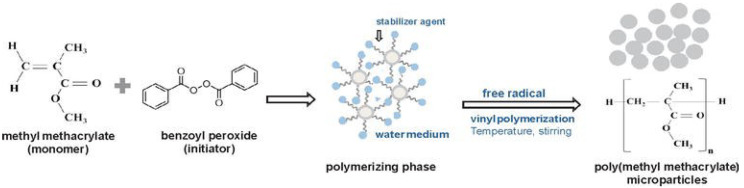
Chemical reaction of PMMA by cold curing process [8].

Last but not least, in the cold cure PMMA type, the tertiary amine initiator, such as dimethyl para-toluidine, was added to the cold cure PMMA, which triggers benzoyl peroxide degradation, and this cold curing process does not require thermal energy. Figure 4 shows the chemistry of the resin has a conversion process from monomer (MMA) to polymer (PMMA) by activation of the tertiary amine of PMMA (polymer) + benzoyl peroxide (initiator) and MMA (monomer) + dimethyl-para-toludin. In the first step of MMA monomer suspension of polymerization, benzoyl peroxide acts as an initiator to interact with the monomer and is equal to two of the water. A soluble water-stabilizer is also helpful to obtain smooth and uniformly sized spherical microparticles.

This causes free radicals to be released, allowing polymerization to occur. Despite a high degree of polymerization resulting in solid physical features, incomplete polymerization and inadequate adaptability are still the key problems in the strength properties of this material. The limitations of the product yield through this polymerization technique are weak bond strength and expensive materials for equipment needed for inactivation [3].

The usage of PMMA as a denture has grown exponentially in recent decades due to its superior characteristics, as mentioned before. Nonetheless, the main concern for PMMA in constructing conventional dentures is the release of residual methyl methacrylate monomer, which is a free monomer when constructed by heat polymerization technique. Although recent studies show only minimal leaching (approximately 5.4 wt%) that occurs in PMMA monomer and is a rare occurrence, this monomer leaching [9] that is released from the PMMA material can also promote allergic reactions, such as denture stomatitis, oedema, and mucosal ulceration, which are made profound by the adhesion of microbes, such as *Candida albicans* and *Staphylococcus aureus* [4,10]. This material is also prone to change in dimensional stability due to error introduced during the manufacturing process within various procedures, such as separating media application, post-dewaxing operation, consistent polymer to monomer ratio, trial closure method, and curing cycle leads to the porosity of the denture. When porosity exceeds 11% from the initial composition of the material, the mechanical and aesthetic properties of the material deteriorated physically, thus providing irregular surfaces for the reservoir of microorganisms, such as *Candida albicans* [10].

## 4. Common Microbial Challenges Associated with PMMA in Conventional Denture

### Denture Stomatitis 

**Figure 5:**
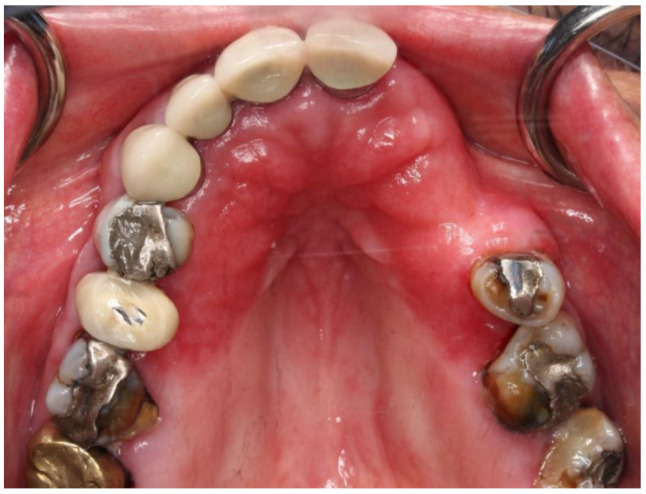
Clinical manifestation of *Candida albicans* sp. fungal infection characterized by the redness (erythema) on the denture base adaptation (saddle) area. In this case, the disease was precipitated by the Sjogren Syndrome due to the low quantity and quality of saliva.

**Figure 6:**
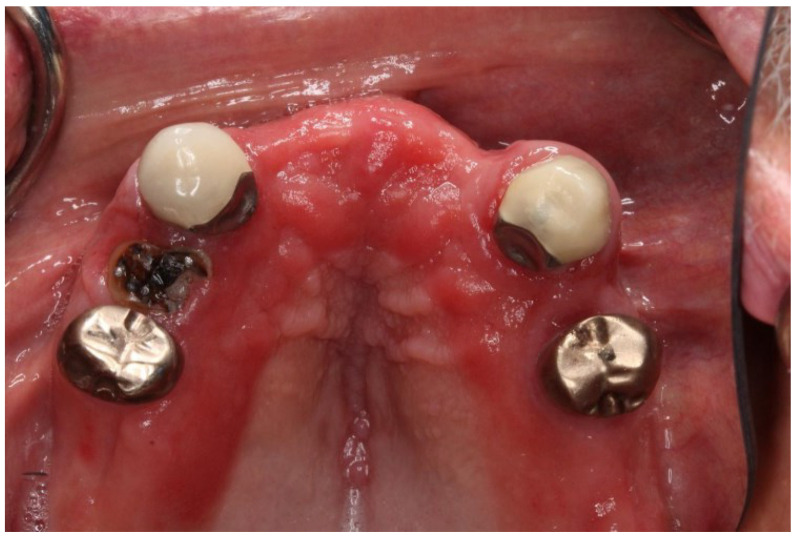
Clinical manifestations of *Candida albicans* sp. fungal infection characterized by the redness (erythema) on the denture base adaptation (saddle) area. In this case, it was aggravated by poor oral hygiene, periodontal disease, and the presence of an untreated root stump.

Denture stomatitis can be divided into three main types. Denture stomatitis Type 1 has localized areas of inflammation, possibly caused by trauma or the denture itself being too tight. Type 2 denture stomatitis, the most common presentation seen in long wearer dentures appears with generalized erythema covering the denture-bearing area (Figure 5) and Type 3 denture stomatitis, known as inflammatory papillary hyperplasia, will usually affect the hard palate or the alveolar ridges (Figure 6) [11].

There are various common causes of denture stomatitis. *Candida albicans* sp. has been one of the main factors in the aetiology of DS and is thought to be responsible for 90% of cases of denture stomatitis. A variety of bacteria, including *Staphylococcus* sp.*, Streptococcus* sp*., Fusobacterium* sp., and *Bacteroides* sp. can be involved [12]. Candida’s position shifts when the immunological balance between the host and the fungus shifts from commensal to parasite and may also act as an opportunistic infection [13]. *Candida* sp. must first adhere to diverse host cells, either directly with the aid of immobilized adhesins, such as integrins or cadherins, or indirectly with the help of other microbes [14]. Adherence proteins such as Amyotrophic Lateral Sclerosis type1-7 (ALS1-7), Amyotrophic Lateral Sclerosis type 9 (ALS9), and Hyphal Wall Protein (HWP1) aid in the adhesion of yeast cells to the host surface. The interaction of ALS proteins with abiotic materials is influenced by hydrophobicity, which contributes to biofilm development, for example, on prosthetic devices. Next, cells will penetrate the tissue via stimulated endocytosis and active penetration in the mouth cavity. *C. albicans* secretes a number of hydrolytic enzymes, including aspartic proteinases, a phospholipase, and a lipase, before penetrating the cellular barriers that will digest the invading cell’s membrane and surface molecules. Secreted aspartic proteinases were already investigated more extensively because they enable the host cell invasion and counteract the host immune system by degrading Immunoglobulin G (IgG) heavy chains, C3 protein, collagen, and fibronectin. Hence, these findings have proven the tissue irritation caused by the host’s weak defence mechanisms. The presence of perfect Candida growth circumstances thus resulting in the fungi being able to colonize the acrylic resin creating favourable conditions for adhesion and proliferation [12].

Moreover, the risk of developing this oral mucosal disease in patients who wear dentures ranges from 36.7% to 65% [15,16]. Continuous and repetitive denture use without periodic tissue rest, damage on the abutment and residual tooth, reduced salivary flow due to chronic diseases, such as Sjogren Syndrome or certain medication, denture age, immunocompromised patient with systemic chronic diseases, such as poorly controlled diabetes mellitus, and harmful habits, such as smoking, are all risk factors for this disease. Furthermore, the rough and hard denture’s fitting surface may also act as a reservoir and aid in the adhesion of microorganisms to the denture and enhancement of biofilm formation.

Poor denture hygiene encourages pathogenic germs to proliferate in dental plaque on denture fitting surfaces, and there is a definite correlation between poor denture hygiene and denture stomatitis lesions. If the denture is worn continually, especially at night, Candida can form a biofilm on the mucosa beneath it. A lower pH creates a relatively anaerobic environment, which encourages Candida growth. Saliva is unable to clean the denture-bearing area, allowing harmful bacteria to flourish. Poor denture hygiene also aided the growth of pathogenic germs in the dental plaque on the denture fitting surfaces. There is a definite correlation between poor denture hygiene and poor denture fit due to inflammation in the denture-bearing area [17].

There are many ways to treat denture stomatitis. One of them is by using antifungal medications that are available in various forms, such as topical application (Amphotericin B, Nystatin, Econazole) to minimize palatal irritation [18] and its benefit is enhanced if used with improved denture hygiene. Next, patient also should learn the correct denture hygiene technique. Effective active methods for cleaning dentures are thorough brushing of the denture in combination with a non-abrasive proprietary paste, which should be carried out after every meal. Moreover, it is also advisable to soak dentures in 0.1% aqueous chlorhexidine or warm soapy water every night to remove microbial plaque on the denture [19].

## 5. Pathogens in Denture Stomatitis

### 5.1. Candida sp. (Candida albicans)

*Candida albicans* is a Candida species that may grow on various surfaces in many ways. *Candida albicans* is by far the most common isolated Candida species found in denture stomatitis presentation, followed by *Candida glabrata* [17]. Candida has the ability to bind with oral mucosa and is composed of key elements in resistance toward the oral cavity’s host clearance systems which function as protective reservoirs, preventing Candida from being washed away by saliva or dislodgement pressures. The capability of this yeast to adhere to mucosal cells, transform from single cell to filamentous form, secrete enzymes, such as aspartyl proteinase and phospholipase, and produce biofilms are all factors that contribute to its pathogenic action [20] *C. albicans*, on the other hand, has a strong desire to stick to the acrylic resin used to make dentures, and the acrylic resin has particular properties, such as hydrophobicity, that aid adherence as a key stage in biofilm development [21]

### 5.2. Staphylococcus aureus

*Staphylococcus aureus* is a Gram-positive bacteria with golden pigment characteristics based on its name ‘Aureus’. It also lives in a facultative anaerobe environment and can withstand high salt, extreme pH, and temperatures. Moreover, *Staphylococcus aureus* also produces many virulence factors and most of it is β–lactamase. *Staphylococcus aureus* can also adhere to a wide variety of oral surfaces, including dental prostheses, which is a non-shedding oral surface. These bacteria can also cause diseases by inducing pyogenic inflammation and producing toxins, such as exfoliative toxins A and B, which can dissolute the epidermal desmosome which causes fine sheets of skin to be peeled off and reveal the moist red skin beneath. Based on a few studies, they also found colonies of *Staphylococcus aureus* from the hard palate are arranged in small clusters obtained from the site of denture stomatitis. These colonies are identified as *Staphylococcus aureus* based on several tests and PCR amplification of Nuc and Coa genes, such as catalase-positive, Pastorex Staph Plus positive, and clumping factor-positive oxidase-negative. A delayed coagulase reaction was observed after a 23-h incubation in the tube coagulase test. Moreover, some studies found that Staphylococcus Aureus from denture wearers have caused greater activation of monocytes than from non-wearers but were less prone to phagocytosis, which suggests stronger virulence factors possessed by *Staphylococcus aureus* [22]. Furthermore, recent evidence suggests that *Staphylococcus aureus* strains are equally and frequently isolated from older persons presenting with denture stomatitis as asymptomatic denture wearers [23] Therefore, from these recent findings, we can conclude that *Staphylococcus aureus* may also contribute to the occurrence of denture stomatitis due to its adherence ability to prosthetic materials and its strong virulence factor [19].

### 5.3. Streptococcus mutans

*Streptococcus mutans* is a common microorganism found on denture surfaces, and if incubated simultaneously with *Candida albicans,* it may occupy the binding sites and might be able to promote yeast adhesion. The in vitro adherence mechanisms of *S. mutans* and *C. albicans* can contribute to understanding these organisms’ behavior in the dental plaque. The interaction of these microorganisms is generally considered as mutual in a combined culture [24] Conventionally, the adherence and maturation of *strep mutans* is regarded as the initial step for oral biofilm formation and distinct adherence mechanisms, which can lead to candidiasis resistance to antifungal therapeutic agents. In complex biofilms, such as those found in the oral cavity, the ability of yeast to agglutinate with bacteria can be mediated by species interaction within the biofilm and external factors, such as saliva, oral hygiene, and exposure to antimicrobial agents. In-vitro evidence showed that despite the Candida–bacteria interaction, emerging studies have investigated that *S. mutans* species can also contribute to patients with candidiasis or yeast-related diseases [25].

### 5.4. Fusobacterium nucleatum

*Fusobacterium nucleatum* is a Gram-negative anaerobe that plays a role in producing plaque and biofilm in the oral cavity. *F. nucleatum* is a well-known “bridging” organism required for the sequential succession of colonization events in oral polymicrobial communities due to its capacity to develop physical contact with Gram-positive and Gram-negative species. It has the ability to link early commensal colonizers with late invaders, mainly composed of periodontal pathogens. As a result, it plays a critical role in the succession of genera in oral polymicrobial communities. Fusobacteria can form direct physical associations with eukaryotic microorganisms, such as fungus, and interact with bacteria. The coherence of *F. nucleatum* and *Candida albicans* has been widely established. It has been linked to the colonization of the oral cavity by *Candida albicans*, which was the main bacteria for the cause of denture stomatitis as stated by Jabra-Rizk et al. in 1999. Such inter-kingdom interactions could be crucial for *C. albicans*’ persistence as part of the microbes within the host and contribute to the advancement of polymicrobial infections under specific circumstances [26]. Therefore, a better understanding of the interacting cellular components should aid in developing new targeted therapies to block adherence. This mechanism could play a key role in polymicrobial pathogenesis involving these two organisms.

## 6. Development of Digital Denture

Computer-aided design and manufacturing (CAD-CAM) systems are being widely used to design and to fabricate fixed, implant, and removable prostheses. Rapid technological advancement in artificial intelligence, data acquisition, rapid prototyping, and digital imaging such as cone beam computed tomography (CBCT) has supported the growth of digital manufacturing methods in dentistry. The manufacturing and fabrication process of dentures derived from the CAD-CAM system was found to be less porous, the most accurate, and with a fast-manufacturing time [27].

Digital dentistry using CAD-CAM technology and high precision 3D printers and scanners can improve dentures’ fit, aesthetics, and functional components, while reducing costs and labour and thus increasing efficiency and manufacturing outcomes. In digital dentures particularly, polymers would be of particular interest because of their ease of fabrication and desirable properties. The ability to design and produce dentures digitally opens the entire range of biomedical polymers for the utilization of material of choice in digital dentistry. The advent of new polymer materials with increased biocompatibility, durability, elasticity, and increased aesthetic appeal and cost-effectiveness offers significant advances for removable prosthetics [27,28].

## 7. Antimicrobial Properties on Digital Denture

One of the elements influencing microbial colonization on denture surfaces and the colour stability of the denture materials could be surface roughness [29] According to the current investigation, milled resins and quickly prototyped resins have comparable surface roughness. According to a study, conventional dentures have a rougher surface area than 3D-printed dentures. This surface substance is essential because it facilitates bacterial adhesions. Rough surfaces may encourage the growth of microorganisms [30].

They are frequently made of poly-methyl methacrylate (PMMA), a porous polymer that is more prone to the build-up of microbial biofilms than conventional dentures [31] The process of forming a biofilm starts with microbial adhesion. It is controlled by the pellicle adsorption of surface-bound salivary proteins and mucins [32].

## 8. Contemporary Modification on Denture Base Material

The summary of materials that have been introduced to improve the conventional and digital denture are compiled as tabulated in Table 1.

Some of the materials have been proven to help reduce bacterial colonization and also improve the overall mechanical properties of the materials. Materials that have helped to improve the denture, particularly biological properties, include Titanium Dioxide (TiO_2_), Graphene-Silver nanoparticles (G-Ag NP), Silver nanoparticles (NAg), graphene, 2-methacryloyloxyethyl phosphorylcholine (MPC) and dimethylaminohexadecyl methacrylate (DMAHDM), Surface Pre-reacted Glass-ionomer (S-PRG), food preservatives, including zinc oxide, potassium sorbate, sodium metabisulfite, nano-silver loaded zirconium phosphate, Phytoncide, Silicon Dioxide nanoparticles (SiO_2_ NP), quaternized N, N-dimethylaminoethyl methacrylate (DMAEMA), and also probiotics [33].

One of the nanoparticles that has been shown to increase the strength of dentures is Titanium Dioxide (TiO_2_). The composite mixture was created by successively adding 0.2 percent, 0.4 percent, 0.6 percent, 1 percent, and 2.5 percent weight-based amounts of TiO_2_ nanoparticles to PMMA. Gram-negative and positive bacteria, fungus, and other microbes are only a few of the microorganisms it has been shown to be active against. Additionally, the papers showed that there is antibacterial activity, and it is environmentally friendly. In addition, TiO_2_ has recently become more well-known because of its apparent high stability, catalytic function, availability, white colour, efficiency, and inexpensive cost. TiO_2_ is corrosion-resistant, non-toxic, and chemically inert. It also has a high refractive index. More research indicates that additional mechanical and biocompatibility tests should be carried out to extend the manufactured dentures for clinical usage [34].

Graphene-Ag nanoparticles are a substance that can be utilised to enhance 3D printed dentures (G-Ag NP). Radio-frequency Chemical Vapour Deposition (RF-CCVD) was used to create the composite of graphene silver nanoparticles (G-Ag Np). The physical and mechanical characteristics of PMMA were improved by the inclusion of graphene and silver nanoparticles. It has been demonstrated that silver fillings have an antibacterial effect. To ascertain whether the higher ratio of free monomers or the added fillers produce the antibacterial effect, additional investigation is required. With *Candida albicans* (the most common cause of prosthetic stomatitis) and other microorganisms that might cause oral infections, they intend to broaden the analysis by adding to the antibiotic spectrum [35].

A new kind of NAg solution (NS)/polymer methyl methacrylate denture base specimens (NS/PMMA) was created by synthesising silver nanoparticles (NAg) solution and combining it with acrylic acid and methyl methyacrylate (MMA) monomer. A widely used form of antimicrobial nanomaterial that has demonstrated a broad spectrum, high efficiency, strong antibacterial activity, and relatively good biocompatibility is easily accessible. Additionally, it is a simple, inexpensive method that has a great chance of developing into an antibacterial treatment for removable partial or complete dentures. It is unquestionably an appropriate material and can be utilised for the population of elderly individuals [36].

Nano-graphene (nGO) is another material that had been introduced to improve the properties of the denture. To clean and sterilize the nano-graphene nanoparticles (nGO) powder, 70% ethanol was used in the washing process. In order to prevent thermal damage to the nGO during heat-induced polymerization, chemically activated PMMA materials were chosen. nGO was added to PMMA powder at weight-relative doses of 0.25 g, 0.5 g, 1.0 g, or 2.0 g. It uses carbon-based nanoparticles to provide sustained, long-term antimicrobial-adhesive activities, without sacrificing mechanical qualities (nGO). Future studies should include novel approaches such functionalizing nGO with PEG, salinization, or carboxyl group to combat the inhomogeneous dispersion of nGO in composite [37].

Moreover, MPC (2-methacryloyloxyethyl phosphorylcholine), a protein repellent that hinders *C. albicans* from adhering to surfaces of denture, was added to other materials to improve the qualities of PMMA denture base materials. Dimethylaminohexadecyl methacrylate (DMAHDM) is also made up of antibacterial agents using the contact-killing method. When these positively charged substances come into contact with a negatively charged cell membrane, the result is a ruptured cell and cytoplasmic leakage. Less biofilm colony-forming units are produced when these ingredients are combined. This demonstrates that adding antibacterial properties and protein repelling chemicals will help conventional dentures to avoid developing denture stomatitis [38].

Next, The PMMA denture base can also be infused with food preservatives including sodium metabi-sulfite, potassium sorbate, and zinc oxide. By generating reactive oxygen species, ZnO promotes cell damage and apoptosis Reactive Oxygen Species (ROS). Two weak acid preservatives, Potassium Sorbate and Sodium Metabisulfite (PS and SM), can stop biological metabolism by accumulating protons inside of the microbial cells, however PS and SM also cause lipid peroxidation, which damages the cell membrane. Additionally, the release of sulphur dioxide molecules has been connected to the sulfur-containing compound SM. Disulfide bonds in protein structures can be broken by these substances, killing enzymes, and causing cellular damage. Therefore, it has been demonstrated that these effects of food preservatives on the denture base also have antibacterial capabilities on the denture base materials [39].

Phytoncide possessed antibacterial properties by weakening the cell wall, inducing bacterial self-degradation due to the disintegration of cell membranes, or interfering with the respiratory metabolism of bacteria. Therefore, the phytoncides that are generated from phenolic compounds may help to reduce microbial activity. Additionally, many of the secondary metabolites that plants generate stress different bacteria. Microorganisms will be impacted by this stress, and this occurrence is understood to be a plant defence mechanism. Allelopathy is a condition that influences ecological systems [40].

By inhibiting bacterial attachment to the denture base and having a self-cleaning effect, the extra layer of film created by SiO_2_ nanoparticles also aids in improving PMMA characteristics. The water molecules in micro-nano scale hierarchical structures are highly polar. It has a potent adsorption effect on dirt that is adhered to the surface, making it simple for the water to roll off the dirt from the sample surface. From a different angle, titanium dioxide further functions as a photocatalyst that would damage the bacterial cell membrane by creating reactive oxygen species, resulting in lipid peroxidation and cell death [41,42,43] As a result, the equilibrium of essential oils, such as K+, Na+, Ca2+, and Mg2+, will be disrupted [44].

Last but not least, *lactobacillus* produces antifungal peptides that might disrupt the fungus’ membrane or render its cytoplasm inactive after consumption, which enhances the qualities of denture materials. These probiotics inhibited and disrupted the Candida biofilm on the denture base resin. However, their used culture media had no impact on the denture base resin’s surface roughness. This will eventually aid in the prevention of denture stomatitis in people who wear dentures [45].

On the other hand, one of the remaining challenging aspects in modification of pure materials is to improve the fundamental properties without compromising other aspects of it. For example, although adding nanoparticles or fibers can improve the strength of PMMA, this may compromise the aesthetic, such as color or translucency, or increase biocompatibility issues via the leaching of degradation products in the oral cavity. Although the current laboratory modification of PMMA have resulted in encouraging outcomes, there is still needful careful interpretations and analysis prior using modified PMMA materials for clinical applications. The majority of biocompatibility and in vivo performance of modified materials are still questionable and require further clinical investigation and presumably human trials. Further research should focus on understanding the interactions of modified materials at the molecular level, the evaluation of various properties following American Dental Association specifications, and clinical performance in either simulated oral environments or in vivo clinical studies

**Table 1:** Summary on modifications on conventional and digital denture to improve general characteristic properties of the material.

Title	Material	Methodology	Effect	Recommendation
Poly (methyl methacrylate) with TiO_2_ nanoparticles inclusion for stereolithographic complete denture manufacturing—The future in dental care for elderly edentulous patients? [34]	Titanium Dioxide (TiO_2_)	The composite mixture has been obtained through subsequent additions of different amounts of TiO_2_ nanoparticles into PMMA the mixture by the weight of 0.2%, 0.4%, 0.6%, 1%, and 2.5%. Preparation used a combination of titanium tetrabutoxide (1.11 mmol) and dimedone (2.4 mmol) in 100 mL of considered alcohol (methanol, isopropanol) which has been allowed to react for 4 h in the 200 mL Teflon^®^ shaft of a PM100 Retsch^®^ (Retsch GmbH, Haan, Germany) colloid mill (with 25 g of quartz grinding balls of 1 mm diameter) at 250 rpm. The synthesization of TiO_2_ nanoparticles were evidenced by using scanning electron microscopy (SEM). Nanocomposites preparation procedure consisted of adding an appropriate amount of titania nanoparticles into PMMA solution under continuous stirring followed by ultrasound direct mixing in a sealed vial within an hour.	Have a large spectrum of activity against microorganisms including Gram-negative and positive bacteria and fungi. Intrinsically environmentally friendly. Effective antimicrobial activity.	Further studies are needed to document the evolution of thermal and rheological behaviour of the PMMA-TiO_2_ nanocomposites to have a complete image of the influence of the nanofiller content. Further mechanical and biocompatibility tests need to be investigated further.
Titanium Dioxide (TiO_2_) polymethylmethacrylate (PMMA) denture base nanocomposites: mechanical, viscoelastic and antibacterial behavior. [46]	Titanium Dioxide (TiO_2_)	A commercial heat curing PMMA denture acrylic was used (Lucitone 550, Dentsply International Inc., PA, USA). The resin was mixed according to the manufacturer’s instructions and packed into the mold space when the resin mix was in a doughy stage. Different ratios of TiO_2_ NPs were carefully weighed and mixed into the PMMA then tested (1 wt. %, 2 wt. %, and 3 wt. %). Characterization was assessed under the Fourier transform infrared (FTIR, Bruker, TENSOR Series FT-IR Spectrometer, Germany) and Scanning electron microscopy (SEM; FE-SEM-JEOL GSM-6610LV). Mechanical characteristics of the composite PMMA/TiO_2_ NP specimens were measured using a universal nanomechanical tester (Bruker, Campbell, CA, USA). The effects of TiO_2_ NP addition on the thermal behaviour of PMMA were examined using differential scanning calorimetry (DSC)/thermogravimetric analysis (TGA; Model SDT-Q600, TA-Instrument, USA). The PMMA/TiO_2_ nanocomposite specimens were cut into small parts weighing 7 mg, sealed in an aluminium pan, and then heated at 10 °C/min to 600 °C under nitrogen. The thermal behaviour data were obtained by software setup.	Improved in mechanical properties including the microhardness, creep related properties and modulus of elasticity. Improving antibacterial activity (*E. faecalis & P. aeruginosa*) by reducing bacterial adherence to cells.	-
TiO_2_ and PEEK reinforced 3D printing PMMA composite resin for dental denture base applications. [41]	Titanium Dioxide (TiO_2_)	The nano-TiO_2_ was prepared by the hydrothermal method as described by Souvereyns, B. et al. 2013. 34 mL of tetrabutyl titanate was slowly added to 100 mL of 4 mol/L HCl solution and stirred for 2 h. The lower layer of liquid processed using hydrothermal process at 180 °C for 12 h. The PMMA photosensitive resin were mixed according to resin base and nano filler weightage ratio. The mixture was then mechanically stirred at 500 rpm/min for 60 min, followed by ultrasonication at 40 W for 1 h. The mixture was placed in the vacuum oven to remove air bubbles and residue ethanol at 50 °C to remove unwanted materials. Digital Light Projection (DLP) Photocuring 3D printing system (Envision Tech, Gladbeck, Germany) were used to print the samples.	Excellent properties, such as stable chemical properties, good physical and mechanical properties, easy to polish, non-toxic, and antibacterial properties. TiO_2_ enhances the antimicrobial properties of the PMMA resin base.	Further research should focus on microwave light-heat conditions on the light-curing resins. Finite element analysis should be use as part of assessment to see the feasibility of stress of these samples intraorally.
A polymethyl methacrylate denture resin’s flexibility, biocompatibility, and antimicrobial activity are enhanced with graphene and silver nanoparticles. [35]	Graphene-Silver nanoparticles (G-AgNp)	The graphene silver nanoparticles (G-AgNp) composite was synthesized through the radio-frequency catalytic chemical vapor deposition (RF-CCVD) method. The mixture of G-AgNp with the acrylic material was done utilizing 95% ethyl alcoholic solution, at room temperature. Continuous mixing and stirring was performed within 30 min. The material was dried in an oven at 40 °C. Synthesis was performed using a methane.	The addition of graphene and silver nanoparticles to PMMA showed improvements in several physical and mechanical properties of the material, including flexural strength. Silver fillers were shown to exhibit an antimicrobial effect.	Further analysis is needed to determine the exact causative reduction of antimicrobial effect of the material. /-Adding other relevant bacterial / fungi strains particularly *Candida Albican* sp.
Characterization and evaluation of a novel silver nanoparticles-loaded polymethyl methacrylate denture base: In vitro and in vivo animal study. [36]	Silver nanoparticles (Nag)	Nag solution was synthetized and mixed with acrylic acid and methyl methyacrylate (MMA) monomer in order to prepare a new type of Nag solution (NS)/polymer methyl methacrylate denture base specimens (NS/PMMA). Dissolve 2.5 g of solid silver nitrate in 50 mL water followed by centrifugation at specified speed (rpm) and addition of alcohol.	The addition of NAg on PMMA has sufficient mechanical properties for clinical application. Biological properties showed that it possessed antibacterial effect with no cytotoxic effect.	-
Nano-graphene oxide incorporated into PMMA resin to prevent microbial adhesion. [47]	Nano graphene oxide (nGO)	nGO powder was washed with 70% alcohol for cleaning and sterilization purposes. nGO was added according to specific weight in relative to PMMA powder and further underwent sonication for 60 min. PMMA powder was mixed into the liquid at a powder (g) to liquid (ml) at a specific ratio (1.2:1.) and underwent low temperature polymerization afterwards	Addition of nGO increase the flexural strength and surface hardness (0.5 wt% onwards). It shows sustained, long-term antibacterial-adhesive effects.	Functionalization of nGO with PEG, salinization or carboxyl group are recommended to ensure homogenization of aggregation of the samples.
Denture Acrylic Resin Material with Antibacterial and Protein-Repelling Properties for the Prevention of Denture Stomatitis. [38]	2-methacryloyloxyethyl phosphorylcholine (MPC) Dimethylaminohexadecyl methacrylate (DMAHDM).	Combination of 10 mmol of 2-(dimethylamino) ethyl methacrylate (DMAEMA, Sigma-Aldrich, St. Louis, MO, USA) and 10 mmol of 1-bromohexadecane with alcohol (3 g ethanol) in a 20 mL vial. Continuous stirring was done for 24 h at 70 °C.	The addition of MPC and DMAHDM causing decrease value in mechanical properties including flexural strength. The addition of those two biomaterials also promotes antibacterial effect, in this research to *Candida albicans* sp.	Multiple bacterial/ fungal strains should be included in future studies to see the effect on the relevant pathogen that can cause intraoral disease particularly to denture wearers. Long term effect of the addition of biomaterials should be evaluated in the context of biological and mechanical properties.
Heat-cured poly (methyl methacrylate) resin incorporated with different food preservatives as an anti-microbial denture base material [39]	Food preservatives, including zinc oxide, potassium sorbate and sodium metabisulfite	The food preservatives were mixed with the PMMA powder to specific concentrations (0.25% *w*/*w* of ZnO, 1.0% *w*/*w* of PS, and 0.5 % *w*/*w* of SM). The powder and liquid are mixed according to the manufacturer guidelines.	Incorporation of food preservative agent, such as ZnO, PS (Potassium Sulphate), and SM (Sodium Metabisulfite), into PMMA denture-based resin help in improving anti-microbial properties with no significant cytotoxicity exhibited. The addition of the food preservatives did not weaken the flexural strength and modulus in comparison to unmodified PMMA.	-
Surface silanization and grafting reaction of nano-silver loaded zirconium phosphate and properties strengthen 3D-printable dental base composites. [33]	Nano-silver loaded zirconium phosphate (6SNP3)	Surface alteration achieved by modification and salinization of γ-methacryloxypropyltrimethoxysilane (MPS) and grafting reaction of methyl methacrylate (MMA). Denture base resin composite materials were prepared with the ratio of P-6S-NP3 to E-Denture resin in a specified ratio (0, 1:100, 2:100 and 3:100, respectively). The procedure was briefed as follows: the 6S-NP3 or P-6S-NP3 nanoparticles were added to E-Denture resin and underwent mechanical stirring at 500 rpm/min for 60 min. The bubbles were removed by vacuum to obtain well-dispersed nanocomposite resin materials for additive manufacturing process.	Denture base composites displayed better mechanical properties in flexural strength, flexural modulus and impact strength. It also showed that the denture base composite possessed better antibacterial efficacy against *E. coli* sp. than *S. aureus* sp.	-
Novel dental poly (methyl methacrylate) containing phytoncide for antifungal effect and inhibition of oral multispecies biofilm. [40]	Phytoncide	The phytoncide was mixed with the denture base resin liquid monomer at various specific weight percentages; 0% (control), 1.25%, 2.5%, 3.75%, and 5%, respectively, and synthesizing further by sonication for 60 min and stirring for 4 h. Specimens were produced after polymerization into specific shapes were done.	The addition of phytoncide did not alter the general mechanical properties of the denture base, in the aspect of microhardness and flexural strength. Phytoncide exerted antimicrobial effects by reducing the amount of *Candida albicans* sp. colony forming units and biofilm thickness in this study.	Recommendation for more studies done to prove the effect of phytoncide incorporated in PMMA has antifungal properties when incorporated within denture base material.
Superhydrophobic coatings with self-cleaning and antibacterial adhesion properties for denture base. [42]	SiO_2_ nanoparticles	0.2 g, 30 nm SiO_2_ NPs were dispersed into isopropyl alcohol (10 g), tetraethoxysilane (TEOS) (0.3 g) and 3-Glycidoxypropyltrimethoxysilane (KH560) (0.9 g) were added. Next, deionized water (30 µL) and acetic acid (30 µL) were added. The mixture was stirred vigorously for one day (24 h) at room temperature to obtain epoxy functionalized SiO_2_. S1, S2, and S3 coatings were made into 10% solution by mixing three solutions with different ratios and spraying on the substrate.	SiO_2_ micro nanoparticles produce an extra layer that prevents bacteria adhesion to the denture base surfaces and contains a self-cleaning effect.	-
Antibacterial activity, cytotoxicity, and mechanical behaviour of nano-enhanced denture base resin with different kinds of inorganic antibacterial agents. [41]	Titanium dioxide (TiO_2_) Silver-supported titanium dioxide (Ag/TiO_2_) Silver-supported zirconium phosphate (Novaron), Tetrapod-like zinc oxide whiskers (T-ZnOw)	Two weight percentage silanized nano-ZrO2 particles and 4 wt% silanized ABW (Aluminium Borate Whiskers) were mixed with PMMA powder by ball milling for 120 min at the speed of 180 rpm. Other materials, such as TiO_2_, Ag/TiO_2_, Novaron, and T-ZnOw, are also added to the composites with specific 3 wt%. The composites were further mixed with MMA monomer at specific ratio (2:1) powder-to-liquid ratio.	The addition of four antibacterial inorganic substrate reducing the colony forming units of *S. mutans* and *C. albican.* T-ZnOw and Novaron possessed the highest surface hardness and flexural strength in comparison to other groups, respectively.	-
Fabrication of denture base materials with antimicrobial properties. [44]	Quaternized N, N-dimethylaminoethyl methacrylate (DMAEMA)	The heat-polymerizing denture base resin was prepared according to the manufacturer’s guidelines. Next, DMAEMA-OB monomer was added to the monomer of the acrylic resin at 0%, 8%, 10%, and 12%, according to the polymer mass. Following the mixing of the powder and monomer, the dough was placed in a customised stainless-steel mold sandwiched between two steel plates and polymerized for 1 h at 70 °C and 2 h at 100 °C (dry heat), while the mould was crushed with a hydraulic press (20 MPa).	The antimicrobial activity was assessed using three different bacteria which are S. aureus (Gram-positive), E-coli (Gram-negative), and *C. albicans*. DMAEMA incorporated in denture base resin possessed antimicrobial effect towards all 3 different bacteria and fungi strains. Nonetheless, careful consideration on incorporating this material in the denture acrylic base as it may cause more plastic deformation, crack and reduce the mechanical properties of the denture base.	Recommendation for the need to improve the mechanical properties of a denture base composed of antibacterial properties.
Inhibitory effects of *Lactobacillus rhamnosus* and *Lactobacillus casei* on Candida biofilm of denture surface. [45]	Probiotics	Polymethylmethacrylate (PMMA) material specimens as denture base resin were prepared. Acrylic resin specimens were processed according to the manufacturer’s instructions. The flask was submerged in water at 74 °C for 90 min and at 99 °C for 30 min. After deflasking, PMMA disks were equivalent in size. Disks were placed in distilled water at room temperature until used. Next, denture base resin was immersed in the spent culture medium of L. rhamnosus and L. casei during the day and in tap water overnight, and this process was repeated for 30 days.	Both *Lactobacillus rhamnosus* sp. and *Lactobacillus casei* sp. possessed antifungal activity against Blastoconidia and *C. albicans* sp. and inhibit formation of biofilm on the denture base. They also did not affect the surface roughness of the denture base.	-

## 9. Conclusions

In conclusion, various materials and methods have been introduced to improve the biological properties, especially the biocompatibility and biofunctionalization of the denture base. Ideally, the incorporation of a specific substrate must improve the biological activity of the denture base, while improving the overall physical, mechanical, and optical properties. Nevertheless, the potential negative effect on mechanical properties upon improving the biological activities after incorporation of specific substrates, for an example incorporation of Quaternized N, N-dimethylaminoethyl methacrylate (DMAEMA), was carefully considered, as previously discussed in Table 1. Further research should mainly focus on improving the properties of the denture material, particularly the denture base material by incorporation of more inorganic and organic compounds, on a wider range of bacterial and fungal strains. A detailed investigation, particularly on characterization and mechanical properties, also needs to be emphasized together with future studies.
